# Overweight prevalence increases from 2 to 8 years of age among children with immigrant background in a Norwegian multiethnic population

**DOI:** 10.1177/14034948251356059

**Published:** 2025-07-16

**Authors:** Ingun Toftemo, Anja Brænd, Anne K. Jenum, Line Sletner

**Affiliations:** 1 Department of General Practice, Faculty of Medicine, Institute of Health and Society, Norway; 2 General Practice Research Unit (AFE), University of Oslo, Oslo, Norway; 3 Department of Pediatrics and Adolescent Medicine, Akershus University Hospital, Lørenskog, Norway

**Keywords:** Ethnicity, immigrants, child, overweight, obesity

## Abstract

**Aims:**

Little is known about ethnic disparities in childhood obesity in Europe. We explored the development of ethnic differences in overweight (including obesity) from age 2 to 8 years.

**Methods:**

We collected routine weight and height measures for 604 children from the Norwegian multiethnic, population-based STORK Groruddalen pregnancy and birth cohort (52.6% European, 26.8% south Asian, and 20.5% Middle East/north African ethnic background). Using chi-square tests for trend and binominal logistic regression analyses adjusted for sex and maternal factors, we explored the development of ethnic differences in overweight. For children with south Asian origin, we performed body mass index adjustments considering their relatively higher adiposity.

**Results:**

From age 2 to 8 years, prevalence of overweight was stable in European children (13.1% at 2 years, and 15.1% at 8 years). In children with immigrant background, prevalence increased; from 5.4% to 17.2% in children with south Asian origin, and from 16.2% to 35.8% in children with Middle East/north African background (*p* for trend < 0.001 in both groups). At age 8 years, the latter group had almost a threefold higher risk of overweight (odds ratio (OR) 2.89; 95% confidence interval 1.54–5.42) compared with European-origin children, while ethnic south Asian children had a double risk (OR 2.07; 1.19–3.59) after adjustments for body composition.

**Conclusions:**

**From 2 to 8 years of age, prevalence of childhood overweight increased in children with immigrant background but remained stable in ethnic Europeans. To reduce ethnic disparities in health, effective efforts to prevent obesity in children with immigrant background should start very early in life.**

## Background

The prevalence of childhood overweight, including obesity (hereafter referred to as overweight), increased from the 1970s to 2000s in most high-income countries, but levels may later have plateaued in northern and western Europe [1,2]. It is, however, unknown whether this applies to children with immigrant background who constitute an increasing proportion of the populations in high-income countries [3]. In Norway, 21% of the population are immigrants or Norwegian-born to immigrant parents [4]. Repeated cross-sectional studies from Europe show that some groups of children with immigrant background may have higher risk of overweight, compared with the majority population [5-7]. Gender roles, feeding practices, low physical activity, economic factors, and the preference of a chubby baby are suggested to influence childhood overweight in ethnic minorities [8].

Children with overweight have at least twice the risk of overweight in adulthood, compared with normal-weight children [9]. However, thinness at birth and in early childhood may also be a risk factor for type 2 diabetes and cardiovascular disease, particularly if followed by an increase in body mass index (BMI) later in life [10].

BMI is widely used to assess weight status, but it does not distinguish between fat mass and lean mass (primarily skeletal muscle), which may be especially important when comparing weight status across different ethnic groups. Similarly, universal growth references may not be applicable to children of diverse ethnic backgrounds [5,11]. Compared with Europeans, people of south Asian descent typically have a higher fat mass and lower lean mass present from birth [12]. This south Asian ‘thin–fat phenotype’ may partly explain why south Asians are two–four times more likely to develop type 2 diabetes than ethnic Europeans, often developing the disease about 10 years earlier and at a lower BMI [12,13]. Therefore, early identification of overweight risk in children of south Asian descent is clinically important.

A Dutch study showed that the prevalence of overweight declined in children with non-immigrant background, but remained stable in children with Turkish, Moroccan, and south Asian background from 2007 to 2015 [14]. However, European prospective studies longitudinally investigating ethnic differences in childhood overweight from early childhood to pre-pubertal age are lacking. Further, few studies consider the role of body composition in children of south Asian origin.

### Aims

We aimed to (1) investigate whether the development of overweight from early childhood to 8 years of age varied among children of European, Middle Eastern, and south Asian descent in a multiethnic, population-based cohort of mothers and children in Norway, and (2) examine the effects of applying BMI adjustments that account for higher adiposity in children with a south Asian background.

## Methods

### Design and study population

We collected data from the prospective, population-based STORK Groruddalen cohort study, of 823 healthy pregnant women followed through pregnancy [15,16], of which 59% had immigrant background. The Groruddalen area in Oslo, Norway has a population that covers a large span in socioeconomic status and has a high proportion of ethnic minorities. Pregnant women were eligible for the study if they were (1) living in one of three specific city districts in Groruddalen, (2) planning to give birth at one of the two study hospitals, (3) at ⩽20 weeks’ gestation, (4) not having pre-pregnancy diabetes or other diseases requiring intensive hospital follow up during pregnancy, (5) able to communicate in Norwegian or any of the eight languages to which all the information material and questionnaires were translated (Arabic, English, Sorani, Somali, Tamil, Turkish, Urdu, and Vietnamese), and (6) able to give informed written consent. While attending antenatal care at the local child health clinics from 2008 to 2010, women were enrolled at mean 15 weeks of gestation. The women and their children were followed through pregnancy (week 28) and at 14 weeks postpartum.

### Outcome variables

Our main outcome variable was overweight at age 2, 4, 6, and 8 years. Using the age- and sex-specific BMI cut-off values defined by the International Obesity Task Force (IOTF), children were classified with thinness grade 1–4, normal weight, overweight, and obesity [17], merged into ‘overweight’ (including obesity) and ‘normal weight’ (including thinness). We used previously collected growth data from preschool age, and recently collected routine weight and height data up to 8 years’ age from visits at the municipal Child Health Service where virtually all children in Norway attend regularly for vaccinations and check-ups [16,18]. According to national guidelines, trained child healthcare nurses should measure weight to the nearest 100 g, and height to the nearest 0.1 cm without shoes and with light clothes.

We calculated BMI as weight/height^2^ (kg/m^2^). Children’s BMI *z* scores, indicating the number of standard deviations an observation is above or below the mean of a reference population, were calculated according to a Norwegian growth standard [19]. Measurements of BMI *z* score values < −4.0 (*n* = 2) were excluded, as we could not rule out inaccurate measurement notations.

### Covariates

The main exposure variable was ethnicity, defined by the child’s mother or maternal grandmother’s country of birth if that country was outside Europe. We focus on results for the largest ethnic groups: (1) Europe (primarily from Norway and other Scandinavian countries); (2) south Asia (primarily from Pakistan and Sri Lanka); (3) Middle East/north Africa (primarily from Iraq, Turkey, Morocco, Afghanistan, Somalia, and Ethiopia). There were <5 % children of mixed heritage.

Based on the literature and earlier findings, we adjusted for sex, maternal age, and education, as well as maternal pre-pregnant BMI, gestational diabetes, and gestational weight gain [18,20].

*Maternal age at enrollment* was used as a continuous variable. *Education* was categorized as: lower level (primary education or less); middle level (completed high school/upper secondary); and higher level (completed university/university–college education ⩾ 4 years). *Mother’s pre-pregnant BMI* was based on measured height and self-reported pre-pregnancy weight at inclusion and classified as normal weight including thinness (BMI ⩽ 24.9 kg/m^2^), overweight (BMI 25.0–29.9), and obesity (BMI ⩾ 30 kg/m^2^) [15]. *Gestational diabetes* was diagnosed according to the World Health Organization 1999 criteria (fasting glucose ⩾ 7.0 mmol/l and/or 2-h plasma glucose ⩾ 7.8 mmol/l) after an oral glucose tolerance test at 28 weeks’ gestation [21,22]. *Gestational weight gain* was here calculated as the difference between weight measured at 28 weeks’ gestation and self-reported pre-pregnancy weight reported at inclusion, divided into tertiles. Maternal smoking status was considered as a covariate but was left out, as very few of the immigrant women smoked.

*Sample size:* Of 823 women enrolled in early pregnancy, we had valid birth data on 783 singletons live born neonates (supplemental figure, flow chart). We excluded 55 children from ethnic groups with low numbers, as well as prematurely born children, and children with known conditions possibly affecting growth. Thus, we consider the children in our study to be presumably healthy. The final study sample consisted of 604 children from the largest ethnic groups (318 European, 162 south Asian, and 124 Middle East/north African) with growth data from birth and up to 6 and/or 8 years.

### Statistical analysis

Descriptive statistics are given as frequencies, proportions (%), and means with standard deviation (SD). We used chi-square tests for trend when assessing changes in overweight over time within each ethnic group. To investigate ethnic differences in children’s risk of overweight, we performed binomial logistic regression analyses for each time point, using Europeans as reference. As no factors can affect your ethnic origin, there are no real confounders to the association with weight status. Directed Acyclic Graphs were therefore drawn prior to analyses to guide the identification of potential mediators.

The following variables were considered covariates, based on previous studies available variables in the dataset and preliminary analyses: Sex, maternal age, and education, prepregnant BMI, gestational diabetes, and maternal gestational weight gain. A variance inflation factor of <1.5 for all covariates indicated that multicollinearity was not a problem. In the final models, we explored potential interactions with our main exposure variable ethnic origin, by performing stratified analyses and by entering cross-product terms one by one. No significant interactions were observed. Results are shown as odd ratios (ORs) with 95% confidence intervals (CIs). As children with south Asian origin have a higher fat mass for a given BMI than the other ethnic groups, we also performed analyses with positive BMI adjustments of +1.12 kg/m^2^ for boys and +1.07 kg/m^2^ for girls in this ethnic group, according to Hudda et al. [11]. For all analyses we used SPSS version 29.0.0.0 (IBM SPSS statistics, NY, USA). *p* values < 0.05 (two sided) were considered statistically significant.

### Ethical statement

The STORK Groruddalen study was approved by The Regional Committee for Medical and Health Research Ethics for south-eastern Norway (reference number 2015/1035), the Norwegian Data Inspectorate and the data protection officer at the University of Oslo. At enrollment, mothers gave written consent to collect children’s routine weight and height from municipal child health services.

## Results

**Figure 1.**
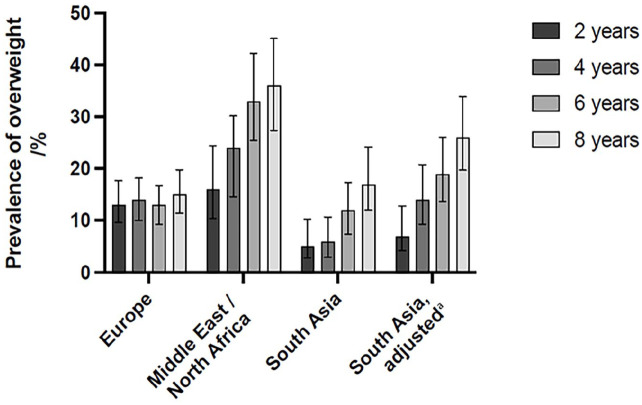
Prevalence of overweight (including obesity) from 2 to 8 years of age in three different ethnic groups. Children with south Asian origin are presented with and without BMI adjustments considering their body composition with relatively higher adiposity.^a^ ^a^Positive BMI adjustments of +1.12 kg/m^2^ for boys and +1.07 kg/m^2^ for girls in children with south Asian origin, according to Hudda et al.[11]. Chi-square test for trend testing if the prevalence of overweight in the three different ethnic groups changed significantly from 2 to 8 years of age. Europe: *p* = 0.3; Middle East/north Africa: *p* < 0.001; south Asia, unadjusted: *p* < 0.001; south Asia, adjusted: *p* < 0.001.

Characteristics of mothers and children are presented in table I. In children of European origin, the prevalence of overweight remained stable: 13.1% at 2 years and 15.1% at 8 years of age (*p* for trend = 0.3; Figure 1). In contrast, in children of Middle East/north African origin, the prevalence was 16.2% at age 2 years, rising steadily to 35.8% at age 8 years. In children with south Asian origin, the prevalence was 5.4% at 2 years of age, rising to 17.2% at 8 years of age (*p* for trend < 0.001 in both groups; Figure 1 and supplemental table). After BMI adjustments, the prevalence in children with south Asian background was 7.4% at age 2 years, and 26.2% at age 8 years. The prevalence of overweight at age 8 years was 18.0% in boys, and 21.8% in girls of the total cohort; for children of European origin 13.6% in boys, and 16.8% in girls; for children with Middle East/north African origin, 27.2% in boys, and 41.9% in girls; and for children with south Asian origin (unadjusted BMI), 20.5% in boys, and 13.4% in girls.

**Table I.** Baseline characteristics of mother–child pairs (*n* = 604).

		Ethnic origin^a^, *n* (%)			
		Total	Europe	South Asia	Middle East/north Africa
		*N* = 604 (100%)	*n* = 318 (52.6%)	*n* = 162 (26.8%)	*n* = 124 (20.5%)
**Mothers**					
Age, mean (SD)		29.9 (4.7)	30.8 (4.4)	28.6 (4.5)	29.4 (5.3)
Education, *n* (%)	Primary education or less	87 (14.7)	12 (3.8)	29 (18.2)	46 (39.0)
	Completed high school/upper secondary	229 (38.6)	99 (31.3)	79 (49.7)	51 (43.2)
	Completed university/college education	277 (46.7)	205 (64.9)	51 (32.1)	21 (17.8)
Pre-pregnant BMI, mean (SD)		24.5 (4.8)	24.6 (4.7)	23.7 (4.2)	26.0 (5.5)
Pre-pregnant weight status, *n* (%)	Thinness	29 (4.9)	10 (3.2)	14 (8.9)	5 (4.1)
	Normal weight	337 (56.6)	188 (59.9)	91 (57.6)	58 (47.2)
	Overweight	143 (24.0)	73 (23.2)	38 (24.1)	32 (26.0)
	Obesity	86 (14.5)	43 (13.7)	15 (9.5)	28 (22.8)
Daily smoking 3 months before pregnancy, *n* (%)		71 (11.8)	63 (20.0)	1 (0.6)	7 (5.6)
Parity, *n* (%)	Para 0	284 (47.0)	169 (53.1)	69 (42.6)	46 (37.1)
	Para ⩾1	320 (53.0)	149 (46.9)	93 (57.4)	78 (62.9)
Gestational diabetes^b^, *n* (%)		186 (31.3)	76 (24.1)	69 (43.1)	41 (34.5)
Gestational weight gain, tertiles (%)	Lowest	198 (33.7)	99 (31.7)	60 (38.2)	39 (32.8)
	Middle	189 (32.1)	103 (33.0)	48 (30.6)	38 (31.9)
	Highest	201 (34.2)	110 (35.3)	49 (31.2)	42 (35.3)
**Children**					
Sex, boy, *n* (%)		306 (50.7)	166 (52.2)	85 (52.5)	55 (44.4)
Birth weight, g (SD)		3493 (516)	3624 (489)	3282 (478)	3434 (531)

a Europeans (primarily from Norway and other Scandinavian countries); Middle East/north Africans, (includes Horn of Africa, primarily from Iraq, Turkey, Morocco, Afghanistan, Somalia, and Ethiopia); south Asians (primarily from Pakistan and Sri Lanka).

b Based on World Health Organization 1999 criteria.

BMI, body mass index; SD, standard deviation.

**Figure 2.**
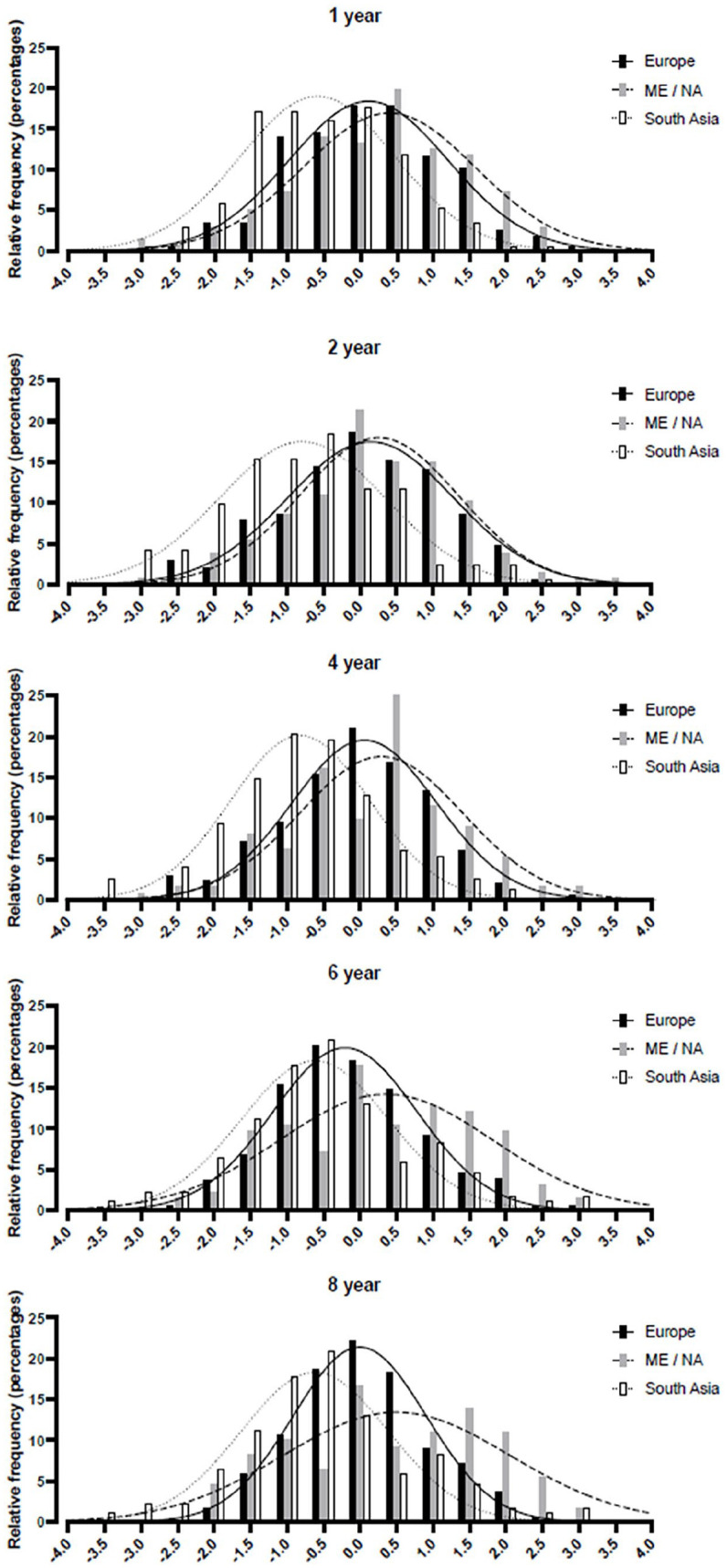
Histograms of BMI *z* scores^a^ at age 1, 2, 4, 6, and 8 years in three different ethnic groups. ^a^Data obtained from a national reference [19]. ME, Middle East; NA, north Africa.

The distribution of children’s BMI *z* scores from age 1 through 8 years showed a shift to the right for children of immigrant descent (Figure 2, table S1). In children with Middle East/north African origin, gradually more children developed overweight, while less children were classified with normal weight as age increased. In children with south Asian origin, more children developed normal weight and overweight, and fewer children were classified with thinness. A shift in BMI class from thinness, through normal weight, and to overweight was most prevalent in children with south Asian background. Of children with overweight at age 8 years, 29% of south Asian children (adjusted BMI), 3% of Middle East/north African children, and 3% of European children had thinness at age 2 years.

Binomial regression analyses showed that the risk of overweight in children with Middle East/north African background at age 2 and 4 years did not differ from European-origin children, when adjusting for sex and maternal factors (Table II). However, at 8 years of age, they had an almost threefold higher risk of overweight (OR 2.89; 95% CI: 1.54–5.42). These estimates changed marginally after adjusting for sex and maternal factors. Children with south Asian origin had lower risk of overweight at 4 years when using universal BMI cut-offs, while similar risk to children of European origin when applying BMI adjustments. From 4 to 8 years of age, the prevalence of overweight increased in children of south Asian descent, so that with universal BMI cut-offs, the risk of overweight did not differ significantly from European children (adjusted OR 1.55; 95% CI: 0.66–2.19) at 8 years. However, after applying BMI adjustments, children with south Asian origin had a doubled risk of overweight (OR 2.07; 95% CI: 1.19–3.59).

**Table II.** Binary logistic regression: Risk of overweight (including obesity) in children with ethnic minority background compared with ethnic European children. Unadjusted and fully adjusted models. Children with south Asian origin are presented with and without BMI adjustments considering their body composition with relatively higher adiposity.^a^

Child’s age	Ethnic origin	Unadjusted OR			Adjusted OR		
					Adjusted for sex, maternal age, education, prepregnant BMI, gestational diabetes, and maternal gestational weight gain		
		OR	95 % CI	*p* value	OR	95 % CI	*p* value
2 years	Ethnic origin (Europe reference)						
	Middle East/north Africa	1.28	0.68–2.40	0.4	1.32	0.60–2.93	0.5
	South Asia	0.38	0.17–0.84	0.02	0.42	0.18–0.98	0.05
	South Asia, adjusted^a^	0.52	0.25–1.05	0.07	0.55	0.25–1.20	0.1
4 years	Ethnic origin (Europe reference)						
	Middle East/north Africa	1.73	0.96–3.10	0.07	1.22	0.58–2.54	0.6
	South Asia	0.38	0.17–0.84	0.02	0.33	0.14–0.76	0.009
	South Asia, adjusted^a^	1.00	0.56–1.80	0.99	0.96	0.51–1.87	0.9
6 years	Ethnic origin (Europe reference)						
	Middle East/north Africa	3.49	2.09–5.81	<0.001	3.21	1.71–6.05	<0.001
	South Asia	0.90	0.50–1.64	0.7	1.07	0.56–2.04	0.8
	South Asia, adjusted^a^	1.69	1.01–2.83	0.05	1.99	1.13–3.51	0.02
8 years	Ethnic origin (Europe reference)						
	Middle East/north Africa	3.12	1.88–5.19	<0.001	2.89	1.54–5.42	0.001
	South Asia	1.17	0.68–2.00	0.6	1.55	0.66–2.19	0.5
	South Asia, adjusted^a^	1.99	1.21–3.26	0.006	2.07	1.19–3.59	0.01

a Positive BMI adjustments of +1.12 kg/m^2^ for boys and +1.07 kg/m^2^ for girls in children with south Asian origin, according to Hudda et. al [11].

OR, odds ratio; CI, confidence interval; BMI, body mass index.

## Discussion

In this population-based cohort of mother–child pairs, we found that children with origin in Middle East/north Africa and south Asia had an increasing trend in prevalence of overweight from 2 to 8 years of age. In contrast, the prevalence remained stable in children of European origin over the same age span. At 8 years of age, children with Middle East/north African origin had an almost threefold higher risk of overweight, compared with children of European origin. When using adjusted BMI values for south Asian origin children, considering their body composition with relatively higher adiposity, the risk of overweight was lower at 2 years while double at 8 years compared with children of European descent. Variations in maternal education, age, pre-pregnant BMI, gestational weight gain, and gestational diabetes did not explain these ethnic differences.

Normal BMI in children varies by sex and age. Most countries use standardized BMI growth curves to monitor growth and establish cut-offs for underweight, normal weight, overweight, and obesity. However, these standards are based on different reference populations and define cut-offs in various ways [23]. In this study we used the international IOTF cut-offs for overweight and sex- and age-specific BMI *z* scores based on a Norwegian reference population [17,24]. Although these cut-offs and growth references are based on a multiethnic population, they may not be equally suitable for all ethnic groups [5]. Ethnicity-specific growth curves or BMI adjustments have suggested to better track healthy growth in children with south Asian background, but there is currently no international consensus on applying specific BMI cut-offs for this group [25].

There are very few prospective cohort studies exploring ethnic differences in childhood overweight in Europe. Hence, our findings of increasing prevalence in children with immigrant background from age 2 to 8 years contribute with important new knowledge. In a Dutch multiethnic cohort (1995–1997), overweight increased from 3 to 15 years of age [26]. In contrast to our findings the increase was similar in children of Dutch and ethnic minority descent (south Asian, Turkish, Moroccan). However, patterns might be different now, and no parental factors besides ethnicity were included in the analyses. We found that the increasing trend of prevalence of overweight in children with immigrant background persisted after adjusting for several maternal metabolic and socioeconomic factors. A study of weight trajectories in children from 9 months to 14 years of age in the multiethnic Millenium cohort living in the UK, showed that children with ‘non-White ethnicity’ in general had an unhealthier growth leading to overweight, compared with children with ‘White origin’ [27]. As this study defined ethnic groups differently from us, it may not be directly comparable to our study. However, ‘south Asian’ children in the British study included children with similar country backgrounds as ours. Their growth trajectory described as ‘late weight gain’ (lower starting point before increase throughout early- and middle childhood) corresponds to and strengthens our findings. Another study of the Millennium cohort showed that children with south Asian background had lower risk of overweight compared with ‘White British’ children at 4–5 years of age [28]. At 10–11 years this difference was no longer significant. However, no trend analysis was reported. This study defined weight class according to BMI *z* scores in a national reference population, and results may have been different if ethnic-specific cut-offs had been used for children with south Asian origin.

Different cross-sectional studies of children with south Asian background living in Europe report various prevalence of childhood overweight in this ethnic group [5]. These differences may partly be due to whether ethnicity-specific BMI cut-offs for south Asians have been applied [5,25]. In our cohort, the prevalence of overweight in children with south Asian origin at age 8 years increased from 17.2% to 26.2% after adjusting BMI values. About 1 in 3 children of south Asian origin children with overweight at 8 years of age (adjusted BMI) were classified with thinness at age 2 years. A growth pattern crossing from thinness to overweight is especially unfavorable as it is associated with increased risk of young-onset type 2 diabetes and more diabetes complications, especially in people with immigrant origin [10]. As south Asians also are more likely to have other risk factors for type 2 diabetes, such as family history of diabetes, more insulin resistance and less physical activity, preventing such a growth pattern is particularly important in this group [13].

Cross-sectional studies from Scandinavia (Denmark, Sweden, and Norway) show that children with non-Western background have more overweight compared with native-born children [29]. The Norwegian Childhood Growth Study, from a national sample of 8-year-old school children, found a higher prevalence of overweight in children with immigrant origin, compared with children with non-immigrant background, except in children with south Asian origin [6]. However, differences were smaller than in our study, possibly partly because the reference group was differently defined. Also, in the other study, the category ‘Asian except south Asian background’ children with east Asian background were grouped together with children of origin in the Middle East countries [6]. These two groups might counterbalance each other, which may explain our finding of a higher prevalence of overweight in children with Middle East origin.

We found no association between sex and overweight up to 8 years of age. This is in line with some other studies from European countries showing minor sex differences in prevalence of overweight during childhood [7,26] In the Norwegian Child Growth Study, a higher proportion of girls than boys had overweight in the non-immigrant group, while there were no significant sex differences in overweight in the group of children with immigrant background [6]. Several national and international studies have shown a higher prevalence of childhood overweight in rural compared with urban areas [1,30]. Thus, ethnic disparities in overweight may be different (possibly smaller) in other parts of the country, compared with our population from eastern Oslo, due to a higher prevalence in the reference population in rural areas.

Strengths of the present study include the population-based cohort design, high attendance rate (74%), and low loss to follow up [15]. Further, validated questionnaires were used when possible, and were translated to eight languages, to support the professional interpreters during the interviews, reducing information bias. At enrollment, 22% of ethnic minority women needed an interpreter. These actions probably reduced selection bias [15]. We have a high-quality data set with extensive questionnaire information, often using clinical data from early pregnancy, making us able to adjust for several important maternal factors. However, several limitations should be noted. We collected growth data from routine visits at the municipal Child Health Clinic where virtually all children in Norway attend regularly for vaccinations and check-ups. This reduces the number of missing, and hence potential selection bias, which is an important limitation in similar studies. Nevertheless, although the routine anthropometric data were collected by trained nurses, they may be less accurate than if measured under standardized conditions. However, this limitation likely applies equally to all ethnic groups. Due to the small sample sizes for some countries of origin, we merged children into three broader ethnic groups, which may introduce bias due to within-group heterogeneity. Additionally, unmeasured covariates, such as fathers’ anthropometric data, may have influenced our results. Lastly, ethnicity is by nature complex and fluid and, although precisely defined, the variable used in our analyses may carry information beyond biological differences. Thus, whether the ethnic differences are mainly mediated by biological differences such as genetic or epigenetic variation, or by unmeasured social, cultural, or psychological disparities is unknown.

## Conclusions

From 2 to 8 years of age, the prevalence of overweight increased among children with immigrant background while it was stable in children of European origin. At 8 years of age, children with Middle East/north African origin had almost threefold higher risk of overweight, compared with children of European origin. When using adjusted BMI values for children with south Asian background considering their body composition with relatively higher adiposity, the risk of overweight was twice that in Europeans.

To avoid acceleration of ethnic disparities in health, prevention of obesity in children with immigrant background should start at an early age. As south Asian populations have an elevated risk of type 2 diabetes, health professionals should be aware that children with south Asian background are often thin of nature [12,13]. Parents may be assured that a relatively low body weight is normal for these children. Extra attention is needed if they start crossing BMI percentiles upwards, even within normal range. More knowledge on drivers, as well as culturally targeted and public health actions, is needed to prevent and treat childhood obesity in immigrant populations.

## Key points

- In children with immigrant background, prevalence of overweight (here including obesity) increased from age 2 to 8 years, while prevalence was stable in children of European origin.
- At 8 years of age, children with Middle East/north African background had almost threefold higher risk of overweight, compared with children of European origin.
- In analyses with adjusted BMI values for children of south Asian descent, considering their relatively higher adiposity, the risk of overweight in this group was twice as much as in children with European descent at 8 years of age.
- To reduce ethnic differences in obesity-related burden and disease, targeted interventions covering children of immigrant descent, as well as broad population-based public health interventions, are urgent.
- Further research is needed on how to tailor interventions suitable to children of different ethnic minorities living in Europe.

## Supplemental Material

sj-docx-1-sjp-10.1177_14034948251356059 – Supplemental material for Overweight prevalence increases from 2 to 8 years of age among children with immigrant background in a Norwegian multiethnic population Supplemental material, sj-docx-1-sjp-10.1177_14034948251356059 for Overweight prevalence increases from 2 to 8 years of age among children with immigrant background in a Norwegian multiethnic population by Ingun Toftemo, Anja Brænd, Anne K. Jenum and Line Sletner in Scandinavian Journal of Public Health

sj-docx-2-sjp-10.1177_14034948251356059 – Supplemental material for Overweight prevalence increases from 2 to 8 years of age among children with immigrant background in a Norwegian multiethnic population Supplemental material, sj-docx-2-sjp-10.1177_14034948251356059 for Overweight prevalence increases from 2 to 8 years of age among children with immigrant background in a Norwegian multiethnic population by Ingun Toftemo, Anja Brænd, Anne K. Jenum and Line Sletner in Scandinavian Journal of Public Health
