# Supplementary figures and images for: Overweight prevalence increases from 2 to 8 years of age among children with immigrant background in a Norwegian multiethnic population
Source: Scand J Public Health. 2025 Jul 16;53(8):889–97. doi: 10.1177/14034948251356059 (PMC12619842; doi:10.1177/14034948251356059)

**Supplementary figure.** Flow chart from inclusion to analyses.

**
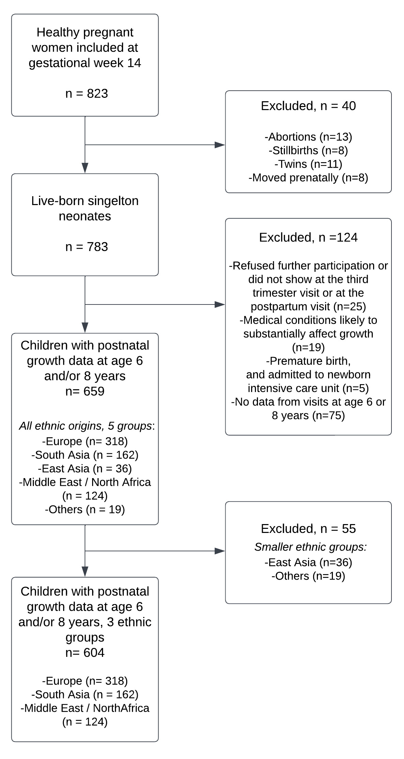
**

Supplement: sj-docx-1-sjp-10.1177_14034948251356059 – Supplemental material for Overweight prevalence increases from 2 to 8 years of age among children with immigrant background in a Norwegian multiethnic population [file sj-docx-1-sjp-10.1177_14034948251356059.docx]
