# Supplementary material for: Overweight prevalence increases from 2 to 8 years of age among children with immigrant background in a Norwegian multiethnic population
Source: Scand J Public Health. 2025 Jul 16;53(8):889–97. doi: 10.1177/14034948251356059 (PMC12619842; doi:10.1177/14034948251356059)
Supplement: sj-docx-2-sjp-10.1177_14034948251356059 – Supplemental material for Overweight prevalence increases from 2 to 8 years of age among children with immigrant background in a Norwegian multiethnic population [file sj-docx-2-sjp-10.1177_14034948251356059.docx]

**Supplementary table.** Weight class at 2, 4, 6, and 8 years of age. Children with South Asian origin are presented with and without BMI adjustments considering their body composition with relatively higher adiposity^a^

|  | **Ethnicity** |  |  |  |  |  |  |  |  |  |
| --- | --- | --- | --- | --- | --- | --- | --- | --- | --- | --- |
| **Weight category** | Europe |  | Middle East / North Africa | | South Asia |  | South Asia, adjusted^a^ | | Total |  |
|  | n | % | n | % | n | % | n | ~~%~~ | n | % |
| 2 years | 267 |  | 105 |  | 148 |  | 148 |  | 520 |  |
| thinness | 33 | 12.4 | 10 | 9.5 | 46 | 31.1 | 15 | 10.1 | 89 | 17.1 |
| normal weight | 199 | 74.5 | 78 | 74.3 | 94 | 63.5 | 122 | 82.4 | 371 | 71.3 |
| overweight | 33 | 12.4 | 13 | 12.4 | 8 | 5.4 | 7 | 4.7 | 54 | 10.4 |
| obesity | 2 | 0.7 | 4 | 3.8 | 0 | 0 | 4 | 2.7 | 6 | 1.2 |
| 4 years | 272 |  | 103 |  | 142 |  | 142 |  | 517 |  |
| thinness | 28 | 10.3 | 11 | 10.7 | 36 | 25.4 | 8 | 5.6 | 75 | 14.5 |
| normal weight | 207 | 76.1 | 70 | 68 | 98 | 69 | 114 | 80.3 | 375 | 72.5 |
| overweight | 30 | 11 | 17 | 16.5 | 6 | 4.2 | 16 | 11.3 | 53 | 10.3 |
| obesity | 7 | 2.6 | 5 | 4.9 | 2 | 1.4 | 4 | 2.8 | 14 | 2.7 |
| 6 years | 311 |  | 117 |  | 157 |  | 157 |  | 585 |  |
| thinness | 32 | 10.3 | 8 | 6.8 | 31 | 19.7 | 7 | 4.5 | 71 | 12.1 |
| normal weight | 240 | 77.2 | 70 | 59.8 | 108 | 68.8 | 120 | 76.4 | 418 | 71.5 |
| overweight | 30 | 9.6 | 26 | 22.2 | 13 | 8.3 | 22 | 14 | 69 | 11.8 |
| obesity | 9 | 2.9 | 13 | 11.1 | 5 | 3.2 | 8 | 5.1 | 27 | 4.6 |
| 8 years | 284 |  | 109 |  | 145 |  | 145 |  | 538 |  |
| thinness | 17 | 6 | 13 | 11.9 | 20 | 13.8 | 3 | 2.1 | 50 | 9.3 |
| normal weight | 224 | 78.9 | 57 | 52.3 | 100 | 69 | 104 | 71.7 | 381 | 70.8 |
| overweight | 35 | 12.3 | 23 | 21.1 | 17 | 11.7 | 23 | 15.9 | 75 | 13.9 |
| obesity | 8 | 2.8 | 16 | 14.7 | 8 | 5.5 | 15 | 10.3 | 32 | 5.9 |

^a^Positive BMI adjustments of +1.12 kg/m2 for boys and +1,07 kg/m2 for girls in children with South Asian origin, according to Hudda et. al.
